# Characterising cerebrovascular reactivity and the pupillary light response–a comparative study

**DOI:** 10.3389/fphys.2024.1384113

**Published:** 2024-08-08

**Authors:** Sierra Sparks, Genevieve Hayes, Joana Pinto, Daniel Bulte

**Affiliations:** Institute of Biomedical Engineering, Department of Engineering Science, University of Oxford, Oxford, United Kingdom

**Keywords:** autonomic nervous system, cerebrovascular reactivity, hypercapnia, pupillometry, smooth muscle, transcranial doppler ultrasound

## Abstract

**Introduction:**

Smooth muscle is integral to multiple autonomic systems, including cerebrovascular dynamics through vascular smooth muscle cells and in ocular muscle dynamics, by regulating pupil size. In the brain, smooth muscle function plays a role in cerebrovascular reactivity (CVR) that describes changes in blood vessel calibre in response to vasoactive stimuli. Similarly, pupil size regulation can be measured using the pupillary light response (PLR), the pupil’s reaction to changes in light levels. The primary aim of this study was to explore the interplay between cerebral blood flow and pupil dynamics, evaluated using CVR and PLR, respectively.

**Methods:**

A total of 20 healthy adults took part in a CVR gas stimulus protocol and a light and dark flash PLR protocol. CVR was calculated as the blood flow velocity change in the middle cerebral artery, measured using transcranial Doppler ultrasound in response to a 5% increase in CO_2_. Multiple PLR metrics were evaluated with a clinical pupillometer.

**Results:**

CVR and PLR metrics were all within the expected physiological ranges for healthy adults. Nine different PLR metrics, assessed through the light and dark flash protocols, were compared against CVR. A significant negative relationship was observed between the latency of the PLR in the dark flash protocol and CVR. No statistically significant relationships were found between CVR and other PLR metrics.

**Conclusion:**

This is the first study to investigate the relationship between cerebral blood flow and pupil dynamics. A significant relationship between dark flash latency and CVR was observed. Future work includes evaluating these relationships using more robust CVR and PLR measurement techniques in a larger, more diverse cohort. Notably, more research is warranted into the PLR using a dark flash protocol and its connection to cerebrovascular function.

## 1 Introduction

Cerebrovascular dynamics are crucial for the maintenance of adequate cerebral blood flow (CBF) to the brain and can be quantified using a metric known as cerebrovascular reactivity (CVR). CVR describes the intrinsic ability for cerebral blood vessels to dilate and constrict in response to vasoactive stimuli, a phenomenon that is largely mediated by vascular smooth muscle cells (VSMCs) that surround arteries and arterioles (Hartmann et al., 2022; Hayes et al., 2022).

CVR can be measured by varying the arterial partial pressure of CO_2_ (PaCO_2_), inducing either hypercapnia (increased PaCO_2_) or hypocapnia (decreased PaCO_2_) through stimuli such as voluntary breathing tasks, gas protocols, or acetazolamide injection (Ringelstein et al., 1992; Liu et al., 2020; Pinto et al., 2021). The concomitant CBF changes can be measured non-invasively using an appropriate imaging modality such as magnetic resonance (MR) imaging or transcranial Doppler ultrasound (TCD). While MR provides CVR measures with relatively high spatial resolution including brain micro-vasculature (Sleight et al., 2021), TCD is a simpler, more widely available and cost-effective alternative that measures blood velocity in single major arteries (McDonnell et al., 2013; Burley et al., 2021). Measurements of CVR are emerging in clinical use to assess cerebrovascular function including in Alzheimer’s disease and dementia (Roher et al., 2011; Suri et al., 2015; Favaretto et al., 2018; Alwatban et al., 2019; Wang et al., 2023), carotid artery stenosis (Ringelstein et al., 1988; Milanlioglu et al., 2022), stroke (Serrador et al., 2000), congestive heart failure (Xie et al., 2005), hypertension (Lipsitz et al., 2000; Barnes et al., 2018).

Smooth muscle can also be found outside of the brain, such as in the iris in the form of sphincter and dilator muscles to control the size of the pupil (Ishizaka et al., 1998). These muscles can be easily assessed using the pupillary light response (PLR, also called the pupil flash reflex). The PLR characterises pupillary size changes to different light conditions (Lerner et al., 2015). These changes are mainly controlled by opposing branches of the autonomic nervous system: whilst the parasympathetic nervous system controls the constriction facilitated by the sphincter muscles of the iris, the sympathetic nervous system controls the dilation facilitated by the dilator muscles of the iris (Winn et al., 1994; Wang et al., 2016; Wu et al., 2020). In response to a light stimulus, the PLR can be categorised into four dynamic phases: response latency, maximum constriction, pupil escape, and recovery (Hall and Chilcott, 2018). Various parameters of the PLR can be extracted from these four phases for further assessment, depending on the application.

The PLR has been used in clinical and research settings as a diagnostic tool for several mental and physical health problems, including acute and traumatic brain injury (Park et al., 2015; Truong and Ciuffreda, 2016; Oshorov et al., 2021), depression (Fountoulakis et al., 1999; Mestanikova et al., 2017; Berman et al., 2018; Miller et al., 2021), diabetes (Lanting et al., 1991; Karavanaki et al., 1994; Yang et al., 2006; Bista Karki et al., 2020), and increased intracranial pressure and intracranial hypertension (Taylor et al., 2003; Chen et al., 2011; Park et al., 2016; Jahns et al., 2019; Romagnosi et al., 2020). Changes in the PLR have also been reported in both preclinical and clinical Alzheimer’s disease cases (Fotiou et al., 2000; Frost et al., 2017; Chougule et al., 2019; Wu et al., 2020), as well as in those identified to have increased risk of developing neurodegenerative disorders (Sparks et al., 2023).

Given that both of these measures appear to be related to a variety of factors including smooth muscle dynamics and function, and additionally show overlapping changes in several pathologies, it is important to investigate their association to better understand pathological mechanisms and their identification. Therefore, this pilot study aims to explore the relationship between the PLR and CVR in healthy adults with no history of cerebrovascular or eye disorders as a means of assessing the interplay between dynamics in the brain and in the pupil.

## 2 Materials and methods

### 2.1 Subjects

We acquired data from twenty healthy subjects with no record of neurological disorders (9F, age range 23–68 years, with a mean of 33.5 ± 11.5 years at the time of acquisition). Inclusion criteria consisted of having no diagnosed cognitive impairment, psychiatric conditions, diabetes, high blood pressure, respiratory, or cardiac health issues. Participants with corrective prescription glasses did take part in the study, but none who had known vision loss and none who had undergone eye or brain surgery. They were also instructed to refrain from consuming caffeinated drinks for 2 hours before the session. All participants provided informed written consent before each session, and the study was approved by the Medical Sciences Interdivisional Research Ethics Committee (MS IDREC) of the University of Oxford’s Central University Research Ethics Committee (CUREC).

### 2.2 Data acquisition

Data acquired in this study included cerebral blood velocities using TCD and a respiratory gas stimulus, and pupil dynamics using pupillometry with light stimuli. For all participants, the sequence of protocols involved the completion of the TCD and gas stimulus first, followed by the dark adaptation and pupillometry protocols, with at least 10 min of time between protocols to change equipment and transfer setups.

#### 2.2.1 Transcranial Doppler ultrasound and gas stimulus

A 2 MHz pulsed transcranial Doppler ultrasound system (7760EN Doppler-BoxX Digital, Compumedics DWL) was used to measure cerebral blood velocities in the middle cerebral artery (MCA). A transmission gel was applied to the transtemporal window of the volunteer and the TCD probe was placed over the gel and secured using an adjustable headset. The location and angle of the probe was changed until a steady blood flow velocity with good signal-to-noise ratio was achieved.

CO_2_ and O_2_ levels in respired air were sampled using a thin nasal cannula placed into both nostrils and an infrared gas analyser (ML206, ADInstruments). The CO_2_, O_2,_ and TCD signals were recorded using a PowerLab 8/35, 8 Channel recorder (PL3508 ADInstruments) and accompanying LabChart Software.

For the gas stimuli, a custom gas delivery system was used to carry out the procedure and accurately monitor physiological parameters throughout it. This system was built in-house at the University of Oxford (Suri et al., 2021). It consisted of a disposable non-rebreathing anaesthetic face mask with a Laerdal bag placed over the participant’s nose and mouth, secured using a head strap. Holes on either side of the mask were covered by unidirectional silicon membranes to allow exhaled air to escape the mask while being sealed during inhalation. A medical-grade respiratory filter was placed at the junction of the disposable circuit and the permanent fixtures to prevent cross-contamination. On the permanent side of the filter, a short length of tubing led to a parallel Y-pieces where respiratory gas mixtures could be delivered one at a time.

Two different levels of inspired gases (medical air and air with 5% CO_2_) were delivered to the face mask at a rate of 15 L/min through unidirectional tubing. The gas cylinders, each fitted with a pressure regulator and flow metres, were operated by hand, following a predefined protocol.

The gas stimulus protocol consisted of a period of baseline measurements of blood flow velocity while the subject breathed normally on medical air for 3 min. After this period, the gas was switched from synthetic medical air (21% O_2_/79% N_2_) to a mixture of 5% CO_2_ balance air (BOC Group, Linde, Surrey, UK) for another 3 min and the subject was instructed to continue breathing normally. Finally, the gas was switched back to medical air and another baseline measurement was taken for 2 min.

#### 2.2.2 Pupillometry and light stimuli

The NeurOptics PLR-3000 hand-held pupillometer was used to measure the pupillary light response (NeurOptics, Irvine, CA). This hand-held pupillometer uses an infrared camera to capture and measure the pupil size, is automated and monocular, and is widely used in clinical practice and research settings.

There were two protocols used to assess the pupillary light response. Before both protocols, subjects had 2 min of adaptation in a dark, quiet testing room, and throughout the pupillometry testing the subject was sat in a chair. Each protocol was done using the NeurOptics PLR-3000 device on one eye at a time.

For each subject, six measurements were performed on each eye, alternating eyes between each trial, starting with the right eye. The first three measurements on each eye, the light flash protocol, were with the positive step-input stimulus, which had a 1 s baseline measurement, a 1 s flash of 50uW white light, and 7 s of post-stimulus measurement, with a 1-min interstimulus interval. The last three measurements on each eye, the dark flash protocol, were with the negative step-input stimulus, which had a 1 s baseline measurement with the 50 uW light on, a 1 s dark flash with the light off, and a 7 s measurement with the light back on, also with a 1-min interstimulus interval. These two protocols were matched to be the opposites of each other for comparison of the positive and negative pulses and the responses they evoked in subjects.

During the measurements, subjects were instructed to keep their eyes wide open and to avoid blinking, and to hold a constant gaze position. The pupillometer was held at a right angle to the subject’s line of sight. All measurements were taken between 09:00 and 16:00 to avoid interference from circadian rhythms.

### 2.3 Data analysis

#### 2.3.1 Cerebrovascular reactivity analysis

The CO_2_, O_2_, TCD, and time courses were exported at a time resolution of 200 Hz and processed using custom scripts in Python 3.10.8. The CO_2_ and O_2_ signals were converted from percent to mmHg using a conversion factor based on the midday pressure reading on the day of each acquisition in Oxford, UK (EODG, 2022). The raw Doppler-BoxX TCD outputs were converted to cm/s using a calibration factor of 202.1 cm/s/V based on the DWL application software and values below 14 cm/s were removed since they corresponded to the bottoming out of the signal.

Two minutes of near-steady state data were extracted from each of the baseline and 5% CO_2_ periods, starting at least 30 s after a transition. The end-tidal CO_2_ (P_ET_CO_2_) peaks were automatically individuated using tools from the SciPy package (Virtanen et al., 2020) to be used as a surrogate for arterial PaCO_2_ (Spano et al., 2013). The mean P_ET_CO_2_, P_ET_O_2_, and TCD blood flow velocity were taken within each segment. The CVR was calculated by dividing the relative change in measured blood flow velocity by the change in the mean P_ET_CO_2_ between the segments as shown in Equation 1, where MCAv_5CO2_ and MCAv_baseline_ are the mean blood flow velocities during the 5% CO_2_ gas and baseline medical air segments respectively, and the P_ET_CO_2 5CO2_ and P_ET_CO_2 baseline_ are the mean end-tidal CO_2_ values within each segment.

$$CVR\left(\%/mmHg\right)=\frac{\frac{{MCA\bar{v}}_{{\;5CO}_{2}}-{MCA\bar{v}}_{baseline}}{{MCA\bar{v}}_{baseline}}}{P_{ET}{CO}_{2\;{5CO}_{2}}-P_{ET}{CO}_{2\;baseline}}\cdot 100$$ (1)

#### 2.3.2 Pupillometry analysis

The time course data from the pupillometry experiments were extracted directly from the NeurOptics PLR-3000 pupillometer in a CSV file format and processed using custom scripts in MATLAB.

The NeurOptics pupillometer automatically calculates several metrics: initial and end pupil diameters, latency, average and maximum constriction velocity, dilation velocity, and time to 75% recovery for each 9 s measurement. All values were averaged across all trials for each participant.

Due to the nature of the PLR protocols, constriction parameters, dominated by the sphincter muscles and parasympathetic nervous system, were only assessed in the light flash protocol, as the dark flash protocol’s constriction amplitude was significantly smaller than that of the light flash protocol where the pupil starts at a larger, dark-adjusted diameter. Dilation parameters, however, were assessed in both the light and dark flash protocols, and have contributions from the dilator muscles and sympathetic nervous system as well as the sphincter muscles and parasympathetic nervous system.

**FIGURE 1:**
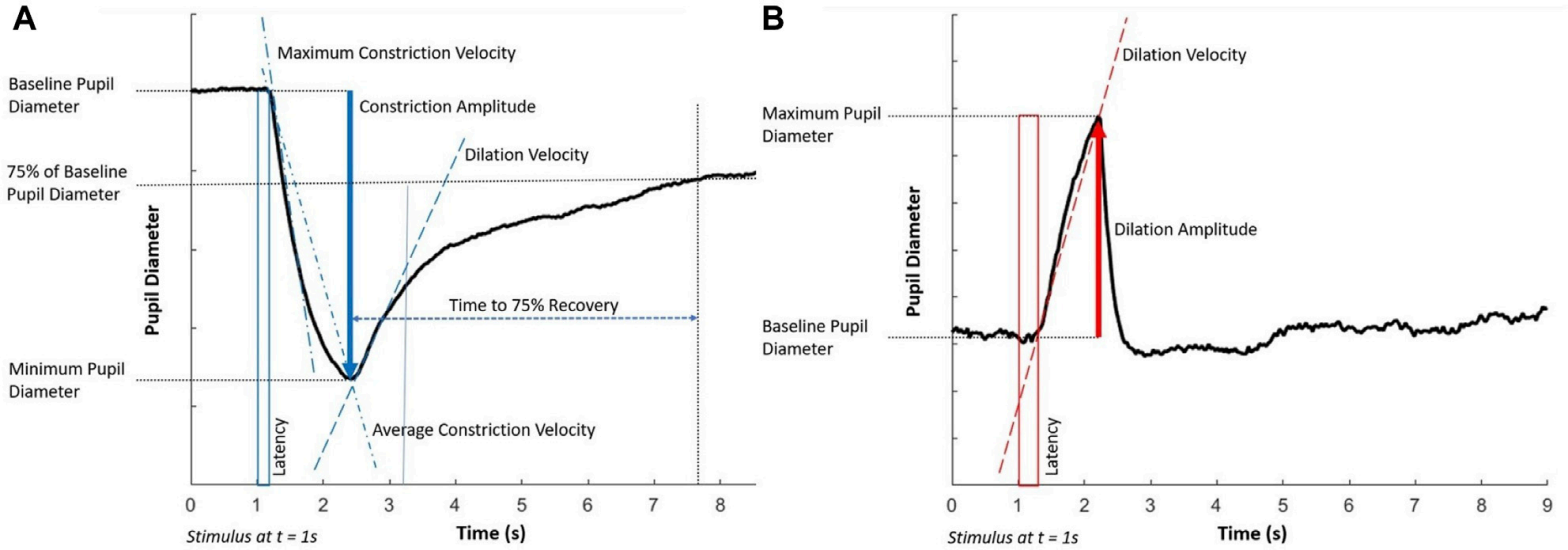
Key components of the pupillary light response to **(A)** the light flash protocol (positive stimulus) and **(B)** the dark flash protocol (negative stimulus). Each stimulus starts at 1 s and lasts for 1 s. Note that the latency in the dark flash protocol (shown in a red box) is longer than in the light flash protocol (shown in a blue box).

For the light flash protocol, the key parameters that were assessed were (a) the average constriction velocity, (b) the maximum constriction velocity, (c) the constriction amplitude, (d) the dilation velocity, (e) the time to 75% recovery, and (f) the latency of the response. For the dark flash protocol, the key parameters assessed were (g) the dilation velocity, (h) the dilation amplitude, and (i) the latency of the response. All these parameters are visually depicted in Figure 1.

#### 2.3.3 Comparative analysis

To identify any statistically significant relationships between the PLR and CVR, we performed linear regression analysis (significance level *p* < 0.05, uncorrected).

## 3 Results

Data from 18 of the 20 subjects were included for analysis. One of the subjects was excluded due to a noisy TCD signal which was likely the result of the probe moving out of alignment with the MCA during the gas protocol. The other participant was excluded due to recent history of smoking, as this could have been a confounding factor to the results.

### 3.1 Cerebrovascular reactivity results

**FIGURE 2:**
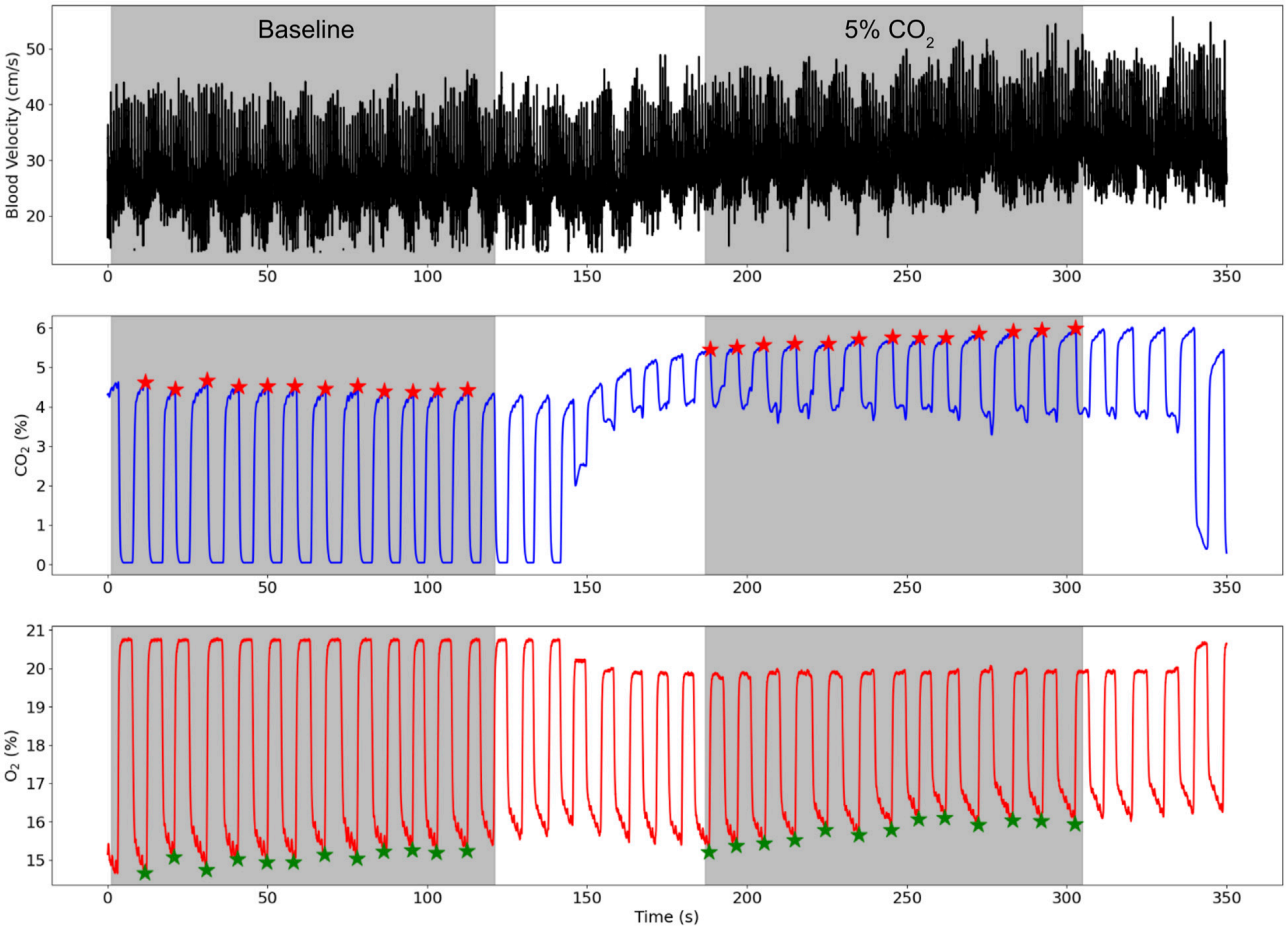
TCD blood flow velocity (cm/s), CO_2_ (%), and O_2_ (%) traces for a representative subject while the subject breathed medical air (baseline) and air with 5% CO_2_ gas. The baseline and 5% CO_2_ periods are shaded in grey and the end-tidal points for the CO_2_ and O_2_ traces are illustrated by red and green stars respectively.

The TCD derived blood flow velocity, CO_2_, and O_2_ traces for a representative subject during the protocol are shown in Figure 2 where the baseline and 5% CO_2_ gas stimulus periods are both highlighted.

P_ET_CO_2_ significantly increased from baseline with a mean P_ET_CO_2_ difference between the 5% CO_2_ hypercapnia period and baseline periods across subjects of 10.01 ± 2.05 mmHg (t-statistic = 9.17, p << 0.01). Similarly, MCA blood flow velocity increased with hypercapnia from baseline with a mean difference across subjects of 9.43 ± 3.24 cm/s (t-statistic = 3.83, p << 0.01). Breathing rates, end tidal points, mean blood flow velocities varied between subjects, but all were within normal and expected ranges (Kirkham et al., 1986; Xie et al., 2005; Peebles et al., 2007; Burley et al., 2021). CVR was calculated using Equation 1 (relative change in MCA velocity compared to the change in P_ET_CO_2_), yielding an average CVR value of 2.90% ± 0.56%/mmHg, across all subjects.

Minimal differences were observed in breathing rate and heart rate between the baseline period and the 5% CO_2_ period. The mean and standard deviation of the breathing rate and heart rate for each period across all 18 subjects are presented in Table 1.

**TABLE 1:** Mean and standard deviation of the breathing rate in breaths per minute (b_r_pm) and heart rate in beats per minute (bpm) of the participants during the baseline period and during the 5% CO_2_ period.

	Mean ± Standard Deviation
Baseline Breathing Rate (b_r_pm)	11.9 ± 5.5
5% CO_2_ Breathing Rate (b_r_pm)	12.0 ± 5.0
Baseline Heart Rate (bpm)	69.1 ± 10.3
5% CO_2_ Heart Rate (bpm)	71.9 ± 6.7

### 3.2 Pupillometry results

**FIGURE 3:**
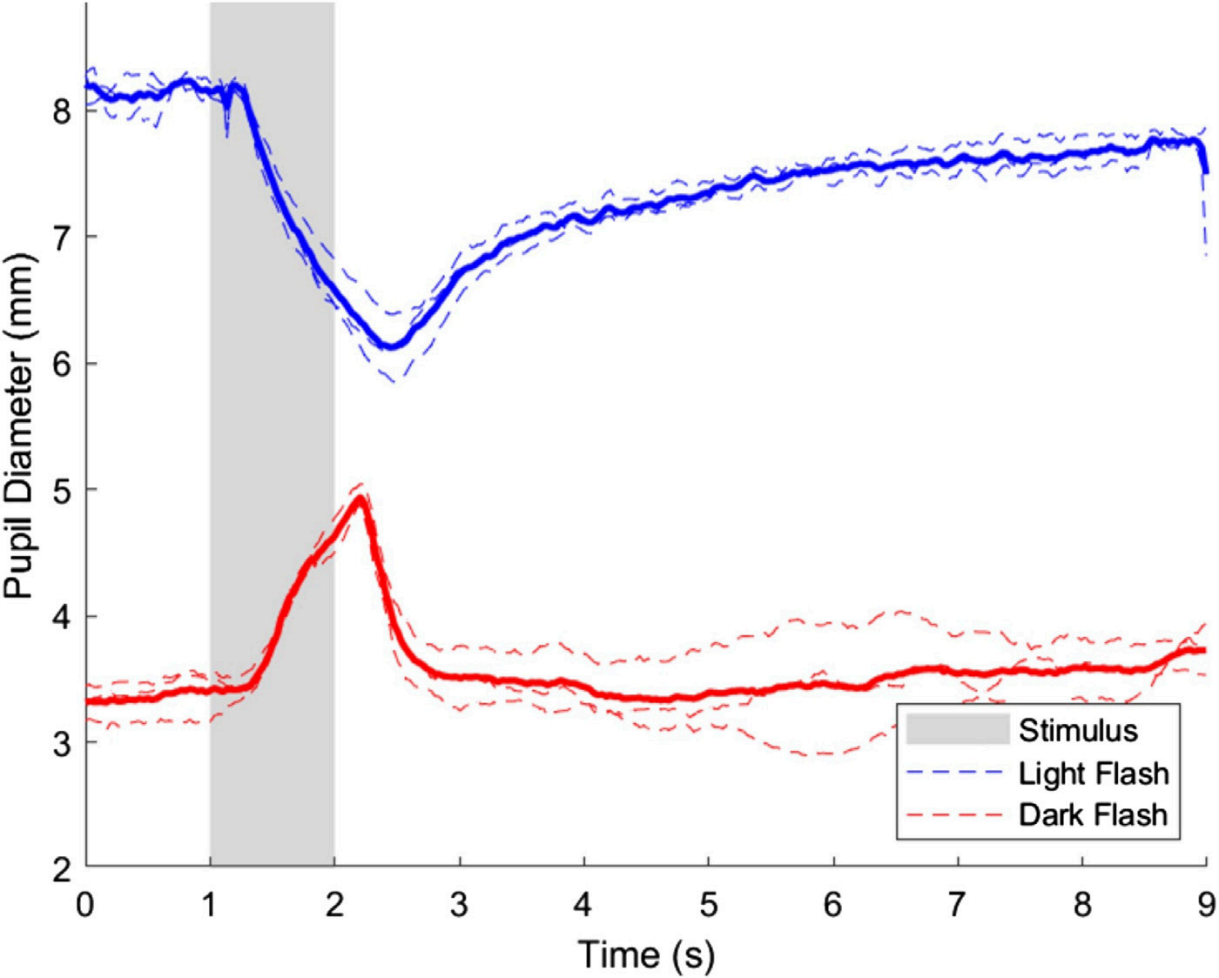
Pupillary light and dark flash response for the right eye of a representative subject. Three trials were performed in the right eye for both the light and dark flash protocols, which are shown on the plot in dashed blue and red lines, respectively. The average response of the light and dark flash protocols in the right eye across trials is shown in a thicker blue and red line, respectively. The stimulus for both protocols started at t = 1s and ended at t = 2s, and is shown in a shaded area on the plot.

The pupillary light and dark responses for the same representative subject in their right eye is shown in Figure 3, where the mean response across all three trials in the right eye is highlighted.

Data from both eyes were collected to ensure that any inconsistencies among subject eyes were noted. However, for the analysis, only the right eye was included for further analysis due to the more complete data among all included subjects. This was also to ensure that averaging across both eyes did not introduce any artefacts.

Figure 3 shows minor differences among individual trials, but the overall pupillary light response characteristics in the right eye were as expected and were comparable to previous studies (Bradley et al., 2011; Martin et al., 2022). The interstimulus interval selected was sufficient for the pupil diameter to return to baseline before subsequent trials. Data from all three trials was averaged to account for minor variations due to hippus and other minor physiological variations that can be expected in assessing pupillary dynamics (Turnbull et al., 2017).

### 3.3 Comparison results

**FIGURE 4:**
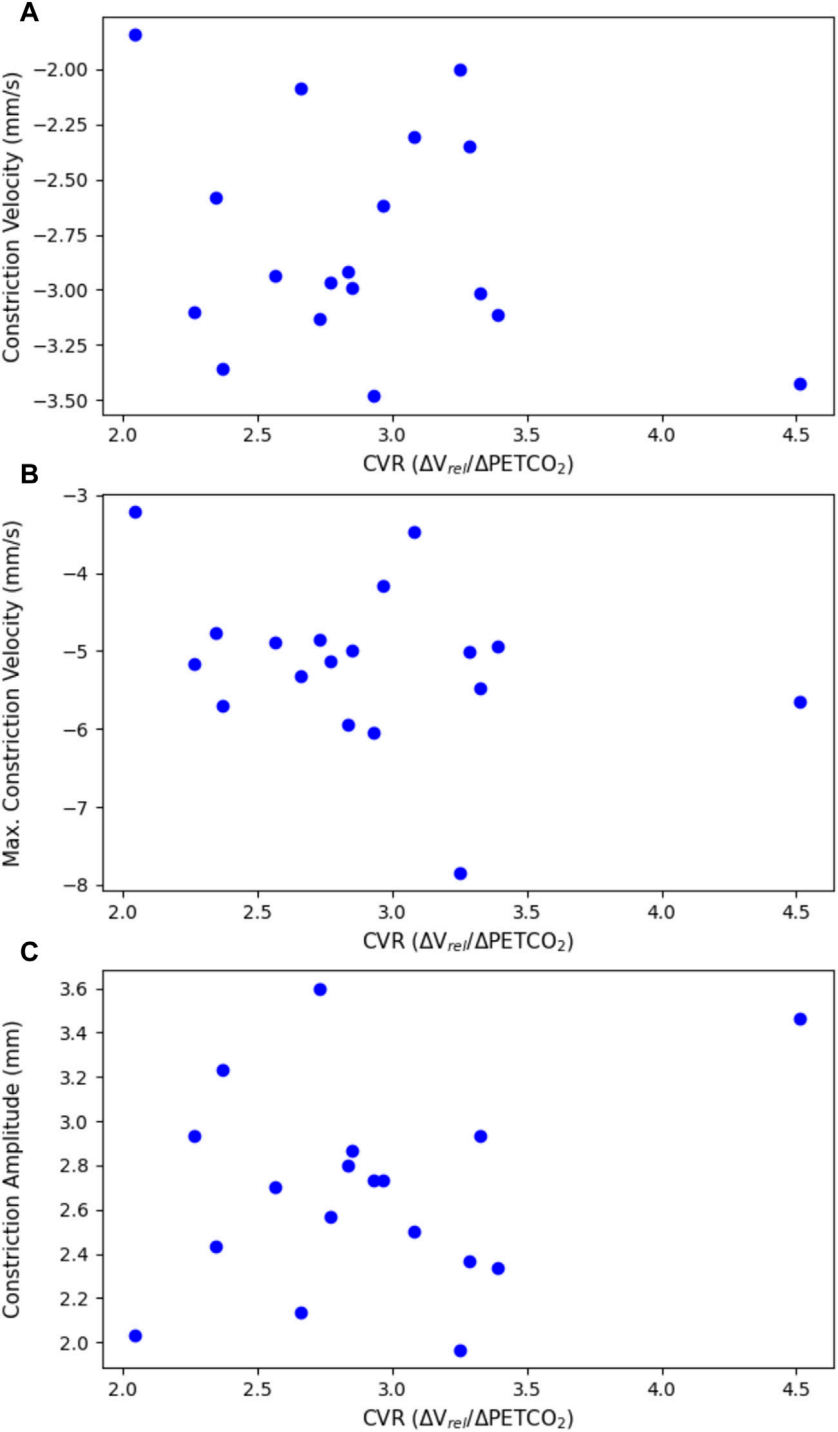
Constriction parameters of the light flash protocol, plotted against CVR. This includes **(A)** the average constriction velocity (*p* = 0.307), **(B)** the maximum constriction velocity (*p* = 0.201), and **(C)** the constriction amplitude (*p* = 0.349), all from the light flash protocol compared to the CVR.

**FIGURE 5:**
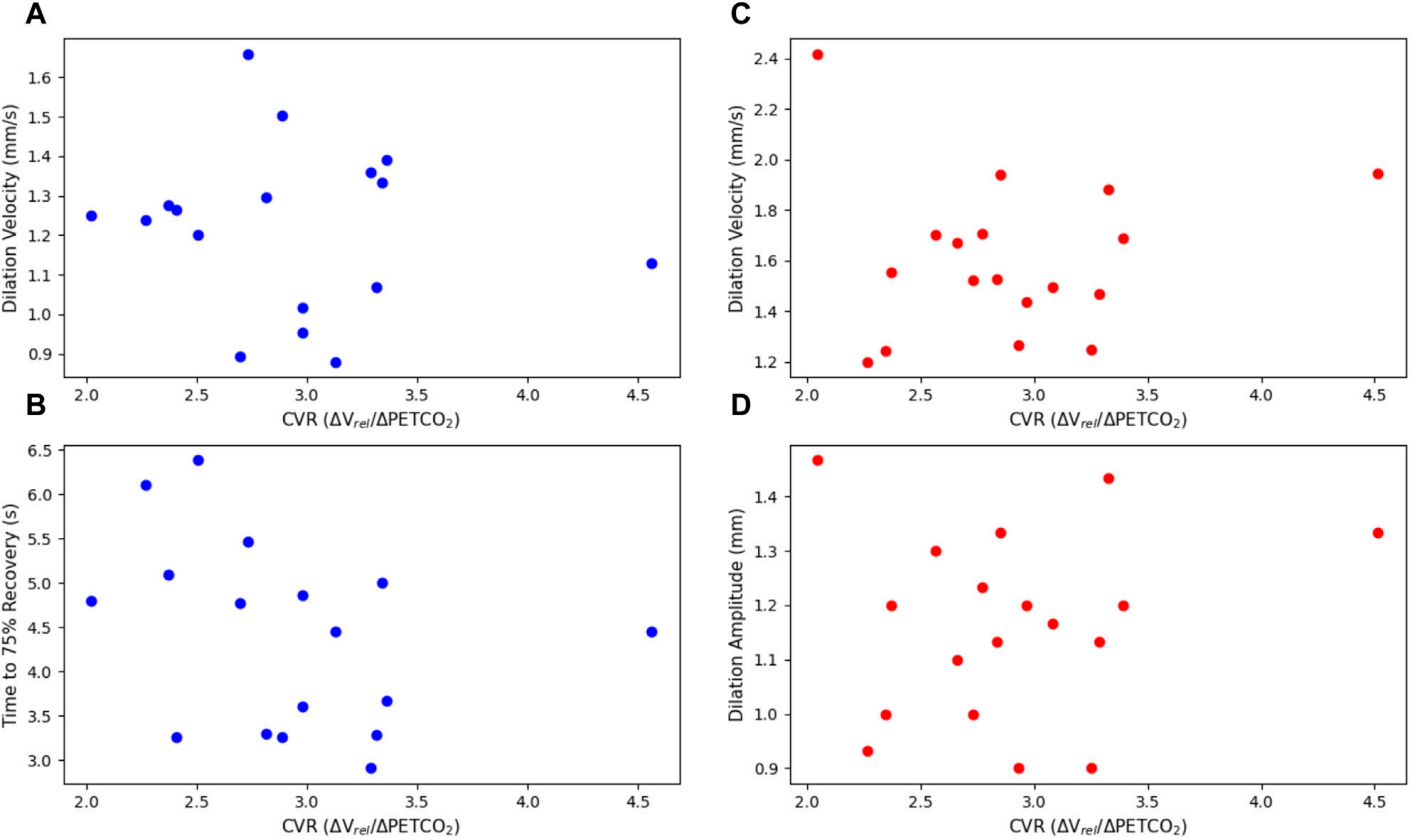
Dilation parameters of the light and dark flash protocols, plotted against CVR. The light protocol **(A)** dilation velocity (*p* = 0.668) and **(B)** time to 75% recovery (*p* = 0.237) are shown on the left in blue. The dark protocol **(C)** dilation velocity (*p* = 0.764) and **(D)** dilation amplitude (*p* = 0.561) are shown on the right in red. Note that one subject is not included in the light flash plots as they did not have a complete dataset for their right eye in the light dilation parameters, due to blinking and other artefacts.

**FIGURE 6:**
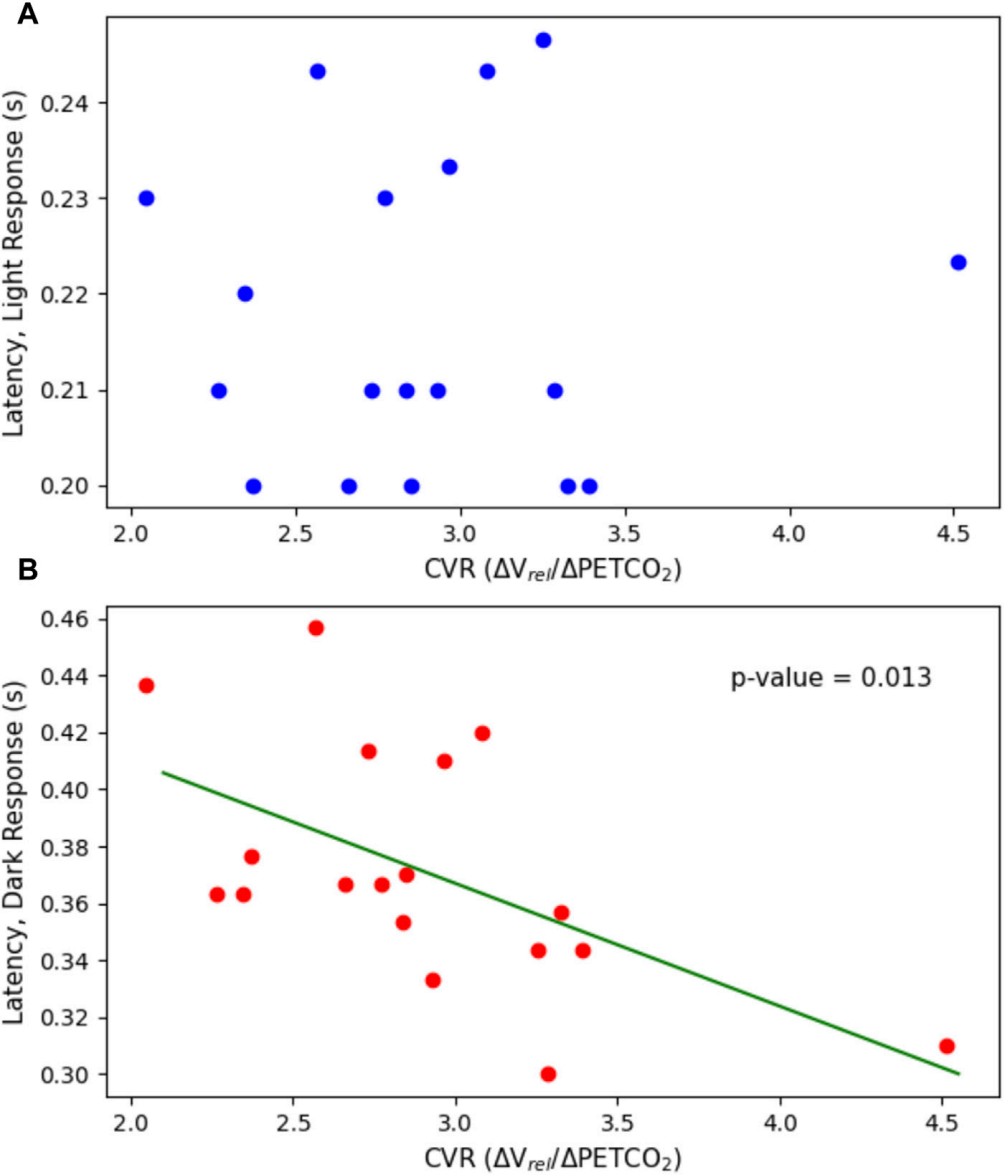
Latency plotted against CVR. This includes the latency in both **(A)** the light flash protocol (*p* = 0.902) and **(B)** the dark flash protocol (*p* = 0.0127) compared to the CVR.

The constriction parameters of the light flash protocol compared to CVR are shown in Figure 4. The dilation parameters of both the light and dark flash protocol compared to CVR are shown in Figure 5. Finally, the latency in both the light and dark flash protocol compared to CVR is shown in Figure 6.

There were no statistically significant linear relationships between the constriction or dilation parameters of the PLR and CVR. There was, however, a statistically significant negative trend (*p* = 0.0127) with the latency in the dark flash protocol and CVR. There was no trend between the latency in the light flash protocol and CVR.

## 4 Discussion

To the authors’ knowledge, this is the first study to provide an analysis of CVR and PLR measurements taken together.

CVR was calculated based on mean MCA blood velocity at baseline and during the inhalation of air with 5% CO_2_ gas. The relative as opposed to absolute change in mean blood velocity between baseline and 5% CO_2_ was used as the measure of interest for the CVR calculation, as this approach mitigates differences in probe location and angle that inherently occurs when collecting TCD data from different subjects. Participants were told to relax and breathe normally throughout the gas stimulus procedure and minimal differences were observed in the breathing rate and heart rate between the baseline and 5% CO_2_ gas periods.

It should be noted that the cerebrovascular response is entangled with physiological mechanisms that affect cerebrovascular function including ventilatory sensitivity, chemoreflexes, and nitric oxide (NO). NO bioavailability has been shown to be a key contributor to cerebral shear-mediated dilation (Van Mil et al., 2002), however some studies have shown that NO synthase inhibition does not influence CVR based on a steady-state CO_2_ stimulus (Ide et al., 2007; Hoiland et al., 2022).

The CVR response to the inhalation of air with 5% CO_2_ can also be impacted by the sensitivity of chemoreflexes including central and peripheral chemoreceptors, central nervous system, and ventilatory response to PaCO_2_ (Ainslie and Duffin, 2009; Carr et al., 2021). Notably, the sensitivity of central chemoreflexes in response to changes in PaCO_2_ can differ between subjects and alter their ventilatory response (Xie et al., 2006). An increase in the ventilatory response to CO_2_ is especially pronounced when CBF is reduced such as in subjects with congestive heart failure and sleep apnea (Xie et al., 2002) and changes in breathing could significantly alter CBF and PaCO_2_ measures. As a result, despite relatively constant breathing rate and heart rate in our study, the CVR response is likely in-part also representative of chemoreceptor and ventilatory sensitivity (Ainslie and Duffin, 2009).

As a result of the complex interplay between these mechanisms, vascular smooth muscle function is unlikely to be the only contributor to the CVR response. CVR may still be a good method for characterising vascular smooth muscle cell function *in-vivo* (Hayes et al., 2022), however the involvement of numerous mechanisms is still poorly understood in humans due to the experimental limitations of isolating independent involvement.

For the PLR analysis, we investigated several parameters relating to both constriction and dilation of the pupil, as there are opposing systems working in both the constriction and dilation phases. Pupillary constriction and dilation are controlled by a variety of physiological mechanisms and neural pathways, including opposing muscles and different branches of the autonomic nervous system. In particular, the parasympathetic/sphincter system dominates the constriction phase with negligible contribution from the sympathetic/dilator system, while both systems contribute to the beginning of the dilation phase (Wang et al., 2016). This means that it is difficult to isolate the specific contributions of smooth muscle alone on various parameters of the PLR, as the smooth muscle dynamics relate strongly to contributions from the sympathetic and parasympathetic nervous systems. Despite the complexities associated with disentangling these relative contributions, assessing specific parameters of the PLR in relation to CVR can potentially provide a valuable insight into the relationship between the eye and the brain.

The average dilation velocity in the light flash protocol (1.22 ± 0.21 mm/s) was consistently smaller than the average dilation velocity in the dark flash protocol (1.61 ± 0.31 mm/s) and this difference was statistically significant (p << 0.001). This could be due to the nature of the protocols. In the light flash protocol, the stimulus first elicits a greater contribution from the sphincter/parasympathetic system to cause constriction, which is likely still active to a certain extent when the dilator/sympathetic system works to dilate the pupil post-stimulus. In the dark flash protocol, however, the stimulus first elicits a contribution from the dilator/sympathetic system, which would explain the larger magnitude of dilation velocity. Additionally, the pupil is only moderately constricted during the light flash protocol when it first begins to dilate, compared to the highly constricted pupil in the dark flash protocol, which would also support a smaller dilation velocity.

The latency of the dark flash protocol (mean = 0.37 ± 0.04s) was consistently larger than that of the light flash protocol (mean = 0.22 ± 0.02s). Conversely, the time to change response directions after the end of the second stimulus, was consistently larger in the light flash protocol than in the dark flash protocol–this demonstrates that the latency in response to a loss of light was larger than that in response to the onset of light.

When comparing the PLR to CVR measurements, most constriction and dilation PLR parameters did not yield statistically significant results. The maximum constriction velocity and the time to 75% recovery showed negative trends associated with CVR, but these were not statistically significant.

Interestingly, there was a significant negative trend relating the latency in the dark flash protocol to CVR (*p* = 0.0127). In contrast, no statistically significant trend was observed with the latency in the light flash protocol and CVR. However, the range of values in latency for the light flash protocol, was significantly smaller than that of the dark flash protocol, which might partially explain the lack of trend. In the dark flash protocol, this statistically significant negative relationship between the pupillary latency and CVR, implies that with a higher CVR, the latency, or time to react to a stimulus change, is smaller. However, if accounting for multiple comparisons, the dark latency falls just outside of statistically significant, therefore additional data and tests are necessary to confirm any significance of the results. Further research is warranted into pupillary parameters of the dark flash protocol, as this protocol has been less studied than the standard light flash protocol.

### 4.1 Limitations and future work

For CVR assessment, we used a 2-point CVR measure as this is the most common method for deriving CVR using TCD (Mitsis et al., 2005; van der Zande et al., 2005; Liu et al., 2020). This strategy assumes a linear relationship between CVR and changes in P_ET_CO_2_. Although it is known that CVR response is in fact sigmoidal in shape (Ringelstein et al., 1988; Bhogal et al., 2014), given our small dynamic range in CVR and P_ET_CO_2_ measurements, we expect that our results fall within the linear range of the sigmoidal curve (Goode et al., 2009; McDonnell et al., 2013). Nevertheless, future research is warranted to further explore more descriptive models of the response of cerebral VSMCs to vasoactive stimuli.

It was also assumed that a steady state was achieved after 30 s of breathing the 5% CO_2_ gas. While we know that P_ET_CO_2_ can continue to increase over even a 10 min period (Poulin et al., 1996), minimal change occurs after the first minute and a long period of breathing air with increased levels of CO_2_ can be challenging for participants. Therefore, to maintain a clinically viable vasoactive stimulus, 3 min was agreed upon as a reasonable upper limit for most volunteers to comfortably breathe 5% CO_2_.

Another possible limitation of the gas protocol was leakage of room air into the face mask during the gas stimulus which was an issue for some participants since the standardised mask did not create a tight seal with all face shapes. Minimal leakage of the 5% CO_2_ gas mixture is visible in Figure 2 by the drops in the CO_2_ trace during the inhales (troughs). Worse leakage was mitigated by using only one ventilation valve which was often one site of room air entry and refitting the mask to ensure no gaps were left around the participant’s nose and mouth.

While a baseline blood pressure measurement was taken for all subjects using an arm cuff to rule out hypertension, continuous arterial blood pressure (ABP) measurements were not acquired in this study. Some studies have shown that changes in ABP, both spontaneous or induced by the inhalation of air with increased CO_2_, can impact CBF velocity in response to vasoactive stimuli and therefore CVR in some adults (Willie et al., 2012; Regan et al., 2014; Howe et al., 2020). However, other studies have shown that even when using air with up to 7% inspired CO_2_, the increase in ABP has minimal effects on MCAv and CVR (Worley et al., 2024). Notably, Dumville et al. also reported that in healthy adults with no vascular disease and intact cerebral autoregulation, the CVR assessment as determined by the relative changes in velocity and P_ET_CO_2_ are independent of ABP provided that the pressure change is contained within the autoregulatory plateau (Dumville et al., 1998). This was echoed by Battisti-Charbonney et al., who showed that the MCAv response to CO_2_ was unchanged by ABP considerations up to a threshold of approximately 50 mmHg, above which both MCAv and ABP appeared to increase linearly with CO_2_ tension (Battisti-Charbonney et al., 2011). However, in patients with pathophysiology such as carotid artery disease, ABP has been shown to significantly alter CVR index calculations in response to inhalation of air with 5% CO_2_ (Dumville et al., 1998). In our study, assessing only healthy adults below that threshold (maximum P_ET_CO_2_ of 46.5 mmHg) when undergoing the gas stimulus, the effects of ABP on our CVR calculations are assumed to be negligible. None-the-less, future studies may benefit from including continuous ABP monitoring (such as by using finger photoplethysmography or more accurately using an arterial catheter) during gas stimulus protocols, especially when investigating pathology.

Furthermore, regional differences in CVR are likely to exist throughout the brain (Leoni et al., 2008; Bright et al., 2009; Pinto et al., 2016), therefore CVR values based on the blood velocity measures in the MCA alone may only be representative of the brain regions supplied by the artery and may not illustrate cerebrovascular function in other regions of the brain.

Lasting cerebrovascular responses triggered during hypercapnic challenges can take additional time to return to baseline post-stimulus, and although the pupillometry was done at least 10 min after the gas stimulus for each participant, there is a small chance that there were still residual hypercapnic effects while the beginning of the pupillometry protocols were being performed. In the future, the PLR data could be collected prior to the gas stimulus. Alternatively, in a larger study cohort, the sequence of protocols could be swapped in half of the study cohort to clarify the PLR without the potential contamination of the after-effects of hypercapnia.

There are also some technical limitations that might have impacted the PLR data collected. Firstly, the frame rate of the NeurOptics pupillometer is low, with only 30 frames per second (i.e., 0.033 s per measurement). When comparing this to the entire range of average latency values in the light flash protocol, which is 0.043 s, this shows that the range of values is comparable to the sampling period of the device. The latency in the dark flash protocol avoids this problem due to the larger magnitude and range of values. With a smaller sampling period, there is potential that a trend could be identified in the light flash latency–this could not be investigated with the limitation of the current equipment. In future experiments, equipment with a higher frame rate should be used to thoroughly investigate any trends between the light flash latency and CVR.

An additional limitation was the assessment of the time to 75% recovery in the light flash protocol. The protocol only included 7 s of recovery time post-stimulus, as it was important to ensure that the entire protocol was short enough so that participants could withstand not blinking for the duration of each trial. In some cases, however, 7 s was not enough time for subjects to recover to 75% of their baseline, initial pupil diameter. When the pupil did not recover to 75% of its initial diameter, no value was reported for this parameter, reducing the number of trials to be included in the average. Additionally, if the subject blinked, the time to 75% recovery and dilation velocity parameters were also not calculated–this also reduced the number of trials included in the analysis for some subjects. In the future, using equipment that can remove blinking artefacts in the data, which would enable a longer recovery time to be included in the analysis, would enable a more confident assessment of dilation parameters in the light flash protocol–especially with the time to 75% recovery, where we would expect to see some higher values recorded.

Although CVR and certain PLR metrics are known to be dependent on age (Feinberg et al., 1965; Eckstein et al., 2017; Peng et al., 2018) and sex (Kastrup et al., 1997; Tallon et al., 2020; Skinner et al., 2023), we did not observe significant differences between ages and sexes. This is likely explained by our small sample size of groups, and as a result statistics could not be confidently performed on the influences of sex and age.

In the future, we plan to increase the dynamic range in vasoactive stimuli, vary the light stimuli for the eye, and improve the imaging resolution for both the blood flow measures and pupil measures. Notably, independently repeating the experiment of the dark flash protocol is necessary to confirm any significance in latency correlating with CVR. This analysis will take place in a larger participant group with a wide range of ages, lifestyle factors, and demographics for a more robust statistical analysis of the interplay between cerebral blood flow and pupil dynamics.

## 5 Conclusion

In this work, we compared the pupillary light response in light and dark flash protocols, to cerebrovascular reactivity assessed using transcranial Doppler ultrasound, to investigate the relationship between dynamics in the eye and brain. We found a significant negative relationship between the latency of the PLR in the dark flash protocol and CVR. No statistically significant relationships were found between CVR and other PLR metrics. This is the first study that has investigated the relationship between cerebral blood flow and pupil dynamics. Future work will incorporate other protocols and equipment, in both pupillometry and in CVR assessment, that might retrieve additional information of interest and further control for confounding factors. Furthermore, a broader range of subjects across age, health, and lifestyle factors will be considered to investigate the validity of these relationships when subject to a larger dynamic range of subjects.

## Data Availability

The raw data supporting the conclusions of this article will be made available by the authors, without undue reservation.
